# Association between depression symptoms and moderately increased levels of the inflammation marker albuminuria is explained by age and comorbidity

**DOI:** 10.1038/s41598-022-12635-1

**Published:** 2022-05-25

**Authors:** Solfrid Romundstad, Torfinn Hynnekleiv

**Affiliations:** 1 Department of Internal Medicine, Levanger Hospital, Nord-Trøndelag Hospital Trust, Kirkevegen 2, 7600 Levanger, Norway; 2 Department of Clinical and Molecular Medicine, Faculty of Medicine, Norwegian University of Science and Technology (NTNU), Trondheim, Norway; 3 Department of Acute Psychiatry and Treatment of Psychosis, Innlandet Hospital Trust, Reinsvoll, Norway

**Keywords:** Depression, Inflammation

## Abstract

The study aimed to examine whether there are associations between depression symptoms and levels of the inflammation marker albuminuria. The 8303 participants in this cross-sectional study were subjects from the second survey of the Trøndelag Health Study (HUNT, Norway). Depression symptoms were assessed by the Hospital Anxiety and Depression Scale (HADS). Logistic regression analysis was performed to estimate the odds ratio (OR) for moderately increased albuminuria (ACR ≥ 3.0 mg/mmol) according to different HADS-depression (D) subgroups and -scores. Unadjusted ORs for moderately increased albuminuria were significantly increased in those with HADS-D ≥ 8 (OR 1.27, 95% CI 1.05–1.54, p = 0.013) and HADS-D ≥ 11 (OR 1.59, 95% CI 1.19–2.14, p = 0.002). After adjusting for age and sex, only HADS-D ≥ 11 was significantly associated with ACR ≥ 3.0 mg/mmol (OR 1.46, 95% CI 1.08–1.98, p = 0.014), and after multivariable adjustments for cardiovascular risk factors and comorbidity, there were no significant associations. However, adjusting for the interaction between age and HADS-D strengthened the association in linear regression models. The positive and significant association between moderately increased albuminuria and symptoms of depression found in unadjusted analyses weakened and disappeared after adjustments. Although individuals with depressive symptoms had albuminuria more often than individuals without such symptoms, and the association seemed to change with age, albuminuria may reflect other comorbidity and inflammation conditions than the depression symptomatology measured in this study.

## Introduction

The immune system may play a part in the pathophysiology of mood disorders^1–3^. Hence, depression has now, for several years, been a research area in the context of immune function and inflammation, like oxidative stress-mediated brain damage^4^, and the relationship between depression and inflammatory biomarkers is well established^5^. Some clinical studies have shown that anti-inflammatory agents in combination with conventional therapy may improve outcomes in mood disorders^6,7^.

One easily accessible and cheap inflammation biomarker is albuminuria. Although moderately increased levels of albumin in the urine, formerly called microalbuminuria, has a high day-to-day variation, persistently increased levels have been well documented to predict cardiovascular (CV) morbidity and mortality in both diabetic and non-diabetic persons^8–10^. Severely increased albuminuria (albumin/creatinine ratio (ACR) > 30 mg/mmol) is probably a direct cause of inflammation in the kidney interstitium, leading to increased progression towards kidney failure, whereas moderately increased levels (ACR 3–30 mg/mmol) are more likely a biomarker of subclinical vascular pathology and atherosclerosis. The link between moderately elevated urine albumin excretion (UAE) and atherosclerosis seems to be inflammation with endothelial dysfunction, leading to increased permeability and leakage of albumin through the vessel wall^11,12^. Today, moderately increased albuminuria appears to be a crucial renal marker for generalized damage and systemic inflammation in the vascular system. Studies support that inflammatory parameters are significantly and independently associated with UAE in prehypertensive, otherwise healthy individuals^13^.

There have not been many studies examining the relationship between depression and albuminuria^14–16^, and to our knowledge, there has never previously been carried out a study in a large population as this health survey. We hypothesised a positive association between moderately increased albuminuria, reflecting inflammation, and levels of depression dimensions.

## Results

The Hospital Anxiety and Depression Scale (HADS)-Depression (D) scores had a median value of 3 (interquartile range 1–6). HADS-Anxiety (A) scores had the same median value (interquartile range 2–6). The baseline characteristics of the population showed that those with HADS-D ≥ 8 had significantly higher mean ACR than those with HADS-D < 8 (1.91 mg/mmol v.s. 1.68 mg/mmol, p = 0.030). They had a higher proportion of moderately increased albuminuria (ACR ≥ 3.0 mg/mmol; 13.3% v.s. 10.8%, p = 0.012), and a corresponding result was found in those with HADS-D ≥ 11. However, in addition to increased albuminuria, those with HADS-D ≥ 8 or ≥ 11 were significantly older and had higher BMI, creatinine and lipid levels than those with lower HADS-D scores. They also had more frequently diabetes, hypertension treatment and cardiovascular disease (CVD), had lower education, and were more often smokers and less physically active than those with HADS-D scores < 8 (Table 1).

**Table 1:** Basic characteristics of the study population, stratified by HADS-D subgroups.

Total	HADS-D < 8 n = 6676	HADS-D ≥ 8 n = 1118	HADS-D ≥ 11 n = 343	p value < v.s ≥ 8	p value < v.s ≥ 11
Age*, years	60.2 (11.6)	63.3 (13.4)	63.1 (13.8)	< 0.001	0.002
ACR*, mg/mmol	1.68 (3.03)	1.91 (3.36)	2.04 (3.74)	0.030	0.093
^‡^Median (25–75% range)	0.80 (0.53,1.43)	0.83 (0.53,1.73)	0.83 (0.53,1.97)	0.383	0.558
ACR ≥ 3.00 mg/mmol^†^ (%)	10.8	13.3	16.3	0.012	0.002
SBP*, mmHg	150 (23)	151 (23)	149 (23)	0.040	0.711
DBP*, mmHg	85 (13)	86 (13)	85 (13)	0.710	0.501
BMI*, kg/m^2^	27.9 (4.5)	28.7 (4.8)	28.7 (5.0)	< 0.001	0.008
Waist circumference*, cm	91.0 (11.9)	93.8 (12.9)	94.7 (13.6)	< 0.001	< 0.001
Creatinine*, mmol/l	91.4 (18.9)	93.3 (18.9)	95.2 (21.5)	0.001	0.002
Cholesterol *, mmol/l	6.2 (1.2)	6.3 (1.3)	6.4 (1.3)	0.002	0.005
Triglycerides*, mmol/l	2.02 (1.22)	2.30 (1.50)	2.45 (1.60)	< 0.001	< 0.001
Glucose*, mmol/l	6.2 (2.5)	6.3 (2.4)	6.5 (2.8)	0.145	0.031
Diabetes^†^ (%)	15.4	20.1	20.8	< 0.001	0.015
Smoking daily^†^ (%)	20.2	22.8	24.2	0.050	0.037
Education high^†^ (%)	13.2	6.7	5.8	< 0.001	< 0.001
Hard physical activity^†^ (%)	17.2	12.1	12.5	< 0.001	0.020
Treated hypertension^†^ (%)	63.6	68.9	69.6	< 0.001	0.018
CVD^†^ (%)	19.3	29.8	34.1	< 0.001	< 0.001

Values are mean (± SD) unless otherwise stated. Independent T-test*, Mann–Whitney U statistics^†^ or Chi-square test^‡^ are used in examine differences.

*ACR* albumin/creatinine ratio, *CVD* cardiovascular disease.

There was a significantly higher prevalence of depression symptoms in those with moderately increased albuminuria (ACR ≥ 3.0 mg/mmol) compared to those without albuminuria (ACR < 3.0 mg/mmol), measured by HADS-D ≥ 8 (15.9% v.s 13.2%, p = 0.012) or HADS-D ≥ 11 (6.0% v.s 3.9%, p = 0.002). Stratified by sex, a corresponding result was found in men. In women, a significantly higher prevalence of depression symptoms in those with albuminuria was found only with HADS-D scores ≥ 11 (6.0% v.s 3.6%, p = 0.017) (Table 2).

**Table 2:** Prevalence of depression (HADS-D ≥ 8 and ≥ 11) in subgroups with or without albuminuria (ACR < and ≥ 3.0 mg/mmol), in the total population and stratified by sex. ACR; albumin/creatinine ratio.

HADS-D score	ACR < 3.0 n = 7365 %	ACR ≥ 3.0 n = 938 %	p value
**Total**			
≥ 8	13.2	15.9	0.012
≥ 11	3.9	6.0	0.002
**Men**			
≥ 8	13.7	16.7	0.035
≥ 11	4.2	6.0	0.051
**Women**			
≥ 8	12.7	14.8	0.206
≥ 11	3.6	6.0	0.017

Unadjusted OR for moderately increased albuminuria was significantly increased in those with HADS-D scores ≥ 8 (OR 1.27, 95% CI 1.05–1.54, p = 0.013) and HADS-D ≥ 11 (OR 1.59, 95% CI 1.19–2.14, p = 0.002). However, after adjusting for age and sex, only HADS-D scores ≥ 11 were significantly associated with ACR ≥ 3.0 mg/mmol (OR 1.46, 95% CI 1.08–1.98, p = 0.014), and after multivariable adjustment there were no significant associations (Table 3). Very similar results were found applying HADS-D as a continuous variable.

**Table 3:** Odds ratio (OR) for moderately increased albuminuria (ACR ≥ 3.0 mg/mmol) according to different HADS-D subgroups/scores.

	Total			Men			Women		
	OR	95% CI	p value	OR	95% CI	p value	OR	95% CI	p value
**HADS-D ≥ 8**									
Unadjusted	1.27	1.05–1.54	0.013	1.31	1.02–1.69	0.035	1.20	0.90–1.60	0.207
Adjusted for age (and sex)	1.14	0.94–1.38	0.186	1.23	0.95–1.60	0.116	1.05	0.79–1.41	0.731
Multivariable adjusted	0.95	0.71–1.26	0.700	1.02	0.71–1.47	0.902	0.83	0.50–1.36	0.450
**HADS-D ≥ 11**									
Unadjusted	1.59	1.19–2.14	0.002	1.49	1.00–2.22	0.052	1.69	1.09–2.62	0.018
Adjusted for age (and sex)	1.46	1.08–1.98	0.014	1.46	0.96–2.21	0.074	1.49	0.96–2.33	0.077
Multivariable adjusted	0.97	0.61–1.54	0.894	0.94	0.53–1.68	0.833	0.95	0.42–2.16	0.896
**HADS-D continuous**									
Unadjusted	1.05	1.03–1.07	< 0.001	1.05	1.02–1.08	0.001	1.04	1.01–1.08	0.007
Adjusted for age (and sex)	1.02	1.00–1.05	0.036	1.03	1.00–1.06	0.051	1.02	0.99–1.05	0.280
Multivariable adjusted	1.00	0.97–1.03	0.845	1.00	0.96–1.04	0.922	1.00	0.94–1.05	0.849

Mulitvariable adjusted for age, sex (in the total population), systolic blood pressure, waist circumference, cholesterol, creatinine, cardiovascular disease, daily smoking cigarettes, treated hypertension, diabetes, education, and hard physical activity.

*ACR* albumin/creatinine ratio.

The significant associations disappeared, especially after adjusting for variables such as SBP, smoking, diabetes, CVD, creatinine, and waist circumference. The interaction effect between age and HADS-D was significant in models with HADS-D ≥ 8. The estimates changed significantly in the adjusted models, and the associations strengthened and became statistically significant in the model adjusted for age (and sex) (Supplementary Table 1). However, the interaction effect was not statistically significant and did not improve models with HADS-D > 11 or continuous HADS-D score. Neither did the interaction term sex*HADS improve any of the models, and thus were excluded.

The associations between HADS-A and albuminuria were not significant in any of the analyses (Supplementary Table 2), except for the continuous HADS-A, unadjusted in the total sample (OR 0.97, 95% CI 0.94–0.99, p = 0.006) and in the women (OR 0.96, 95% CI 0.93–1.00, p = 0.040).

After replacing the categorized dependent variable with the continuous ACR, the interaction term age*HADS-D improved the models, especially when HADS-D was included as a continuous variable (Table 4). The associations between HADS-D and ACR became significant also after multivariable adjustments. In individuals younger than 70 years, there seemed to be inversely associations between ACR and HADS-D, although not significant except for HADS-D ≥ 11 in the age group younger than 50 years (Table 5).

**Table 4:** Linear regression models for ACR as continuous dependent variable, according to HADS-D as a continuous score.

	Total			Men			Women		
	β	95% CI	p value	β	95% CI	p value	β	95% CI	p value
**HADS-D continuous**									
Unadjusted	0.015	0.008, 0.022	< 0.001	0.017	0.007, 0.026	< 0.001	0.013	0.004, 0.022	0.005
Adjusted for age (and sex)	0.004	− 0.003, 0.011	0.235	0.006	− 0.004, 0.015	0.247	0.004	− 0.005, 0.013	0.401
+ Interactions Age*HADS (p = 0.001)	− 0.041	− 0.069, − 0.013	0.005	− 0.048	− 0.089, − 0.007	0.023	− 0.030	− 0.069, 0.010	0.140
Sex*HADS (p = 0.175)	− 0.009	− 0.030, 0.011	0.367						
Sex*HADS + Age*HADS	− 0.059	− 0.094, − 0.023	0.001						
Multivariable adjusted	− 0.004	− 0.012, 0.004	0.351	− 0.004	− 0.014, 0.007	0.523	− 0.004	− 0.016, 0.009	0.554
+ Interactions Age*HADS (p = 0.014)	− 0.043	− 0.076, − 0.011	0.009	− 0.048	− 0.093, − 0.003	0.035	− 0.030	− 0.077, 0.018	0.225
Sex*HADS (p = 0.023)	− 0.033	− 0.059, − 0.007	0.014						
Sex*HADS + Age*HADS	− 0.076	− 0.118, − 0.034	< 0.001						

Mulitvariable adjusted for age, sex (in the total population), systolic blood pressure, waist circumference, cholesterol, creatinine, cardiovascular disease, daily smoking cigarettes, treated hypertension, diabetes, education, and hard physical activity, and with the interaction terms age*HADS-D and sex*HADS-D (total sample).

*ACR* albumin/creatinine ratio.

**Table 5:** Age-stratified association of depression and albuminuria in linear regression models with ACR as continuous dependent variable.

	20–49 years (n = 1853) β (95% CI)	50–69 years (n = 3730) β (95% CI)	≥ 70 years (n = 2720) β (95% CI)	p value for interaction age*HADS-D
HADS-D ≥ 8	− 0.023 (− 0.154, 0.107)	− 0.073 (− 0.183, 0.036)	0.048 (− 0.106, 0.202)	0.156
HADS-D ≥ 11	− 0.265 (− 0.483, − 0.048)	− 0.059 (− 0.247, 0.129)	0.128 (− 0.123, 0.379)	0.007
HADS continuous	− 0.006 (− 0.019, 0.007)	− 0.006 (− 0.018, 0.007)	0.006 (− 0.012, 0.024)	0.014

Mulitvariable adjusted for age, sex, systolic blood pressure, waist circumference, cholesterol, creatinine, cardiovascular disease, daily smoking cigarettes, treated hypertension, diabetes, education, and hard physical activity.

*ACR* albumin/creatinine ratio.

The non-responders on HADS data were significantly older, had higher SBP and cholesterol, were more frequent women, had more often diabetes, CVD and hypertension treatment, reported lower education and less hard physical activity, compared to those with HADS data (Supplementary Table 3). The outcome variable ACR was not significantly different between the two samples. Similar results were found on the missing sample of the hard physical activity-variable compared with the responders (data not shown). The non-responders had more risk factors and comorbidity, were significantly elderly and more frequent women. The prevalence with HADS-D ≥ 8 did not differ between the two samples. We also ran sensitivity analyses without “education” and “hard physical activity” in multivariable models, and neither estimates nor significance levels changed.

## Discussion

This study demonstrated a significant positive association between the measured depression symptoms and moderately increased albuminuria. However, the association weakened after adjusting for age and sex, and after multivariable adjustments the significant association disappeared. Therefore, the unadjusted correlation between depression symptoms and albuminuria seems to be explained by other factors causing possible inflammation, like increased CV risk and other comorbidities.

A positive association between albuminuria and depressive symptoms/depressive episodes has been published by Martens et al.^14^. That study also had a cross sectional design, however with fewer participants, and almost 1/3 had diabetes type 2, compared to 16% in our study. Although adjustments of this comorbidity were carried out in both studies, the population from the Netherlands might still have a higher CV risk profile. Additionally, the studies differed in methods regarding depression symptomatology (PHQ-9 versus HADS), and Martens et al. included a clinical interview (MINI) to assess the presence of minor or major depressive episodes, which gives a more diagnostic classification of depressive episodes. HADS is not an instrument for clinical diagnoses, but it identifies seven core depressive symptoms. A literature review, which included 31 studies, concluded that the HADS holds good properties for depression in patient populations in primary care and hospital settings^17^. HADS has been used in several studies, including population-based studies like HUNT^18,19^. Various cut-off levels have been reported from various study populations. Even high scores of HADS-D do not give in itself a specific depression diagnosis, but studies comparing HADS-D with «gold standard», i.e. reference standard diagnostic interviews for a depression disorder, have disclosed reasonable validity^20,21^. The Norwegian version of HADS is found specifically to be validated for the symptoms of anhedonia and psychological distress^22,23^.

Depression and stress related symptoms may be associated with a chronic, low-grade inflammation response, and studies have shown associations between depression and activation of cell-mediated immunity as well as increased oxidative and nitrosative stress^24^. Several factors increase the risk for the development of depression whilst they also are associated with systemic inflammation; psychosocial stressors, poor diet, physical inactivity, obesity, smoking, altered gut permeability, atopy, dental cares, sleep and vitamin D deficiency^24^. Some of these factors are also associated with albuminuria, like physical inactivity^25^, obesity^26,27^ and smoking^28,29^, and were included as confounders in our analyses, and might have been stronger associated with albuminuria than depression symptoms. The strengthened association between the continuous variables HADS-D and albuminuria after including the interaction term between age and HADS may reflect that the relationship between depression and inflammation changes over time^30^. Younger individuals with depression seem to have a negative association with albuminuria unlike the elderly, which could reflect other mechanisms of depression than in the elderly. Additionally, the link between inflammation and depression may be hidden by the association between an adjusted variable and depression, even though the association is mediated by inflammation.

Inflammation as part of atherosclerosis and dysfunction of the small blood vessels in the brain, and a frequent cause of stroke and dementia, is also hypothesized to lead to depression (the vascular depression hypothesis)^31,32^. A significant positive association between vascular dementia and low-grade albuminuria^33^ and between lacunar stroke and albuminuria^34^ was recently published based on the same HUNT material. Our results did not convincingly support the vascular depression hypothesis regarding depression and albuminuria. However, the results may reflect a limitation of HADS in measuring depression more than the true association between depression and inflammation.

There are several strengths of this study. The population-based approach and the high attendance rate make selection bias less likely. The response rate in the albuminuria screening was especially high in the elderly and those with diabetes and treated hypertension^35^. Another strength was that albuminuria analyses were performed in fresh urine samples without long-term storing, in contrast to other studies^36^. Several studies conclude that measuring ACR is a specific and sensitive alternative to twenty-four-hour urine collection in population-based albuminuria screenings^37,38^.

One of the most important limitations is the self-reported symptoms of depression, which may lead to misclassifications. As the HADS questionnaire does not mirror somatic depressive symptoms, i.e. more directly biologically related symptoms, weight changes, fatigue and insomnia, might be undetected. However, these symptoms overlap with several other morbidities, which might be associated with albuminuria, thus somewhat underestimating than overestimating our results. Although HADS-D might not be the best choice of questionnaire to assess certain depressive features in the context of immunometabolic dysregulation, HADS has still shown association with inflammation-variables, e.g. CRP^39^.

Regarding missing data analyses, significantly elderly individuals with several comorbidities were more often not-responders, which could also underestimate the results. There is probably a considerable variability of inflammation within the depressed population, and there are studies that find subtypes of depression which are more inflammation-related^40^. Further, we had no information about medical treatment of depression, where treatment as tricyclic antidepressants could increase CV risk^41^ in contrast to selective serotonin reuptake inhibitors which are associated with reduced CV risk^42^. We had neither information about use of other medications, which could have influenced albuminuria levels, i.e. use of, among others, angiotensin converting enzyme (ACE) inhibitors and angiotensin II blockers (ATB). However, at the time of the second HUNT, the use of ACE inhibitors and ATB in Norway was still low.

Another limitation is the lack of measuring other inflammatory markers. The HUNT study includes a large population and questions about several diseases, and because of limited resources specific inflammation- or biological parameters for most diseases were not included. Neither do we have information about specific psychiatric risk factors and -comorbidities or all other comorbidities in this epidemiological study since comorbidities are very heterogeneous. We therefore adjusted for available risk factors and comorbidities most correlated with albuminuria and depression, as described in previous literature and verified in our bivariate analyses.

Regarding the diagnosis of urinary tract infection (UTI), were those who reported UTI in the previous week excluded from the analyses, but it was not possible to adjust for asymptomatic UTI. This could contribute to a non-differential misclassification that might weaken associations.

Although the association between symptoms of mood disorders and albuminuria was non-significant after multivariable adjustments in this study, there was a significantly increased prevalence of albuminuria in the depressive symptoms sample compared to the non-depressive. Intervention in albuminuria positive individuals with diabetes or hypertension are recommended and include more aggressive treatment of CV risk factors like blood pressure, smoking, hyperlipidemia and overweight^43,44^. So far, there are no recommendations of albuminuria screening in otherwise healthy people, although small studies have found a reduction of CVD by intervention with ACE inhibition^45^. Individuals with depression and elevated peripheral inflammatory markers, included albuminuria, might have benefit of anti-inflammatory agents, such as acetyl-salicylic acid (ASA), statins, ACE inhibitors and ATB to improve the treatment of depression and other mood disorders, leading to reduction of disability, even though there is much more research to be carried out here.

In conclusion, the positive and significant association between moderately increased albuminuria and depressive symptoms found in unadjusted analyses weakened and disappeared after adjustments, indicating that albuminuria is stronger associated with age, other comorbidities, and CV risk factors. A different screening or diagnostic tool for depressive symptoms may also be considered.

## Methods

### Study subjects

During 1995–1997, the second survey of the Trøndelag Health Study (HUNT, Norway), invited all residents in Nord-Trøndelag County ≥ 20 years (n = 93 898), and a total of 70% participated. The population of this county is considered as stable and ethnically homogeneous, and fairly representative for Norway, except there are no large urban areas, and income and education levels are slightly lower than average. The survey comprised questionnaires, which included questions on HADS, CVD (angina pectoris, myocardial infarction and stroke), smoking habits, other lifestyle factors, and a clinical examination. All those with self-reported diabetes mellitus and/or treated hypertension and a 5% randomly selected sample of the total population were invited to deliver three consecutive morning urine samples as a part of the albuminuria screening study. A total of 9598 participants delivered all three urine samples (overall response rate 84%). Of these, 8801 had answered questions with depression and/or anxiety dimensions. More details of the HUNT study design and albuminuria screening have been published previously^35,46^. Those who answered confirmatory to one of the questions about UTI in the previous week, persistent haematuria in the previous year, menstruation at time of urine collection or pregnancy were excluded from the analysis (n = 338). So were also 160 individuals with severe albuminuria (ACR ≥ 30 mg/mmol), leaving a total of 8303 subjects included in the main analysis.

### Clinical examination

The clinical examination included standardized measurement of height, weight, blood pressure and pulse rate. Height was measured without shoes to the nearest centimetre, and weight was measured to the nearest half-kilogram while wearing light clothing without shoes. Three consecutive standardized blood pressure measurements were recorded at one-minute intervals. After a minimum of two minutes of rest, the measurements were performed in the sitting position, using an automatic oscillometric method (Dinamap 845XT; Criticon, Tampa, FL).

### Measurement of signs and symptoms of clinical depression and anxiety

Symptoms of anxiety and depression were assessed by the HADS^17^. HADS consists of seven depression related items (HADS-D) and seven anxiety related items (HADS-A), each with a four-point ordinal scale to describe symptom severity from 0 to 3. HADS response was defined as a completed answer when five or more items on the subscale have been scored. Missing responses among those who filled in 5 or 6 items were replaced based on the sum of completed items multiplied by 7/5 or 6/5, respectively.

### Urine- and blood sampling

The participants included in the albuminuria screening received a unit with three plastic receptacles for three first morning urine samples and three transport tubes, and one envelope for return by mail back to the laboratory. Additionally, the participants received instructions for urine collection, information about the albuminuria-screening and a questionnaire about UTI, haematuria and menstruation.

Blood sampling was carried out whenever subjects attended (i.e. in a non-fasting state). Fresh serum and urine samples were analyzed at the Central Laboratory at Levanger Hospital, on Hitachi 91 Autoanalyzer (Hitachi, Mito, Japan). Details of the laboratory methods have been published^47^. Urine albumin and creatinine were measured by an immunoturbidimetric (anti-human serum albumin, Dako Norway, Oslo) and Jaffé method, respectively. ACR was an expression for urine albumin excretion.

### Statistical analysis

Body mass index (BMI) was calculated in kilograms per meter squared. Systolic (SBP) and diastolic blood pressure (DBP) were included as the mean of the second and third of three measurements. ACR was calculated as [urine albumin (mg)]/[urine creatinine (mmol)], and was defined as the mean of three ACRs. Moderately increased albuminuria was defined as ACR ≥ 3.0 mg/mmol and < 30 mg/mmol. Both HADS-D and -A have positively skewed distributions, and median (interquartile range) was used to describe the centre (and spread value). ACR has a similar distribution, but both mean and median were calculated for this variable. We used the t-test for independent samples, chi-square or Mann–Whitney U statistics to examine baseline differences between two means, medians or proportions.

Logistic regression analysis was performed to estimate the odds ratio (OR) for moderately increased albuminuria (ACR ≥ 3.0 mg/mmol) according to different HADS-D/A subgroups and -scores, and for HADS-D/A as continuous variables. Additionally, linear regression analyses were conducted for ACR as a continuous dependent variable. Since ACR has a positively skewed distribution, the variable was ln-transformed prior to regression analysis in order to approach a normal distribution. A small constant was added before ln-transformation when the value of ACR was zero. Slopes of regression lines, β, with 95% CI and p value were calculated. A priori selected potential confounding factors were used in both bivariate and multivariable adjusted analyses, i.e. age, sex (in the total population), SBP, waist circumference, cholesterol, creatinine, history of CVD (yes/no), daily smoking (yes/no), treated hypertension (yes/no), diabetes (yes/no), education (primary and secondary school [≤ 12 years] and college or university [> 12 years]) and strenuous physical activity (< 1 h/week, ≥ 1 h/week).

A correlation matrix with means/SD and Pearson correlations among nearly all variables in this study is included in the Supplementary Table 4.

Additional analyses explored the potential interaction effects between age and sex (total sample) on exposure (HADS) in the adjusted models.

A small number of individuals had missing data on HADS-D (6%, n = 509) and education (6%, n = 514), while several had missing data on “hard physical activity” (38%, n = 3164). We ran separate analyses on the responders compared to the non-responders regarding (1) HADS- and (2) physical activity-questions. Additionally, we ran sensitivity analyses without the covariables education and physical activity. All statistical analyses were conducted with the Statistical Package for the Social Sciences, version 25.0 (SPSS Inc., Chicago, USA), and the significant alpha criterion was set to 0.05 or above.

### Ethics approval

All participants signed written, informed consent. All surveys, protocols and linkage of date were approved by the Norwegian Data Inspectorate and by the Regional Committee for Medical and Health Research Ethics (according to no. 2009/1193 4.2006.2481). All methods and experiments were carried out in accordance with relevant guidelines and regulations.

## Supplementary Information

Supplementary Information.

## Data Availability

All data generated and analyzed during this study are available from the corresponding author on reasonable request.

## References

[1] Rosenblat JD, Cha DS, Bansur RB, McIntyre RS (2014). Inflamed moods: A review of the interactions between inflammation and mood disorders. Prog. Neuro-Psychopharmacol. Biol. Psychiatry.

[2] Raison CL, Miller AH (2013). The evolutionary significance of depression in Pathogen Host Defense (PATHOS-D). Mol. Psychiatry.

[3] Beurel E, Toups M, Nemeroff CB (2020). The bidirectional relationship of depression and inflammation: Double trouble. Review. Neuron.

[4] Toker S, Shirom A, Shapira I, Berliner S, Melamed S (2005). The association between burnout, depression, anxiety, and inflammation biomarkers: C-reactive protein and fibrinogen in men and women. J. Occup. Health Psychol..

[5] MacGiollabhui N, Ng TH, Ellman LM, Alloy LB (2021). The longitudinal associations of inflammatory biomarkers and depression revisited: Systematic review, meta-analysis, and meta-regression. Mol. Psychiatry.

[6] Bai S (2020). Efficacy and safety of anti-inflammatory agents for the treatment of major depressive disorder: A systematic review and meta-analysis of randomised controlled trials. J. Neurol. Neurosurg. Psychiatry..

[7] Lee C-H, Giuliani F (2019). The role of inflammation in depression and fatigue. Front. Immunol..

[8] Arnlov J (2005). Low-grade albuminuria and incidence of cardiovascular disease events in nonhypertensive and nondiabetic individuals: The Framingham Heart Study. Circulation.

[9] Hillege HL (2001). Microalbuminuria is common, also in a nondiabetic, nonhypertensive population, and an independent indicator of cardiovascular risk factors and cardiovascular morbidity. J. Intern. Med..

[10] Romundstad S, Holmen J, Hallan H, Kvenild K, Krüger O, Midthjell K (2002). Microalbuminuria, cardiovascular disease and risk factors in a nondiabetic/ nonhypertensive population. The Nord-Trøndelag Health Study (HUNT, 1995–97), Norway. J. Intern. Med..

[11] Satchell S (2013). The role of the glomerular endothelium in albumin handling. Nat. Rev. Nephrol..

[12] Stehouwer CD, Smulders Y (2006). Microalbuminuria and risk for cardiovascular disease: Analysis of potential mechanisms. J. Am. Soc. Nephrol..

[13] Navarro-González JF, Mora C, Muros M, Garcia J, Donate J, Cazaña V (2013). Relationship between inflammation and microalbuminuria in prehypertension. J. Hum. Hypertens..

[14] Martens RJ (2018). Albuminuria is associated with a higher prevalence of depression in a population-based cohort study: The Maastricht Study. Nephrol. Dial. Transplant..

[15] Dalui A, Guha P, De A, Chakraborty S, Chakraborty I (2014). Assessment of stress and related albuminuria in caregivers of severe mentally ill persons. Indian J. Med. Res..

[16] Takasaki K, Babazono T, Ishizawa K, Miura J, Uchigata Y (2016). Relationship between diabetic nephropathy and depression: A cross-sectional analysis using the Diabetes Study from the Center of Tokyo Women's Medical University (DIACET). BMJ Open Diabetes Res. Care.

[17] Bjelland I, Dahl AA, Haug TT, Neckelmann D (2002). The validity of the Hospital Anxiety and Depression scale: An updated literature review. J. Psychosom. Res..

[18] Bjerkeset O, Romundstad P, Evans J, Gunnell D (2008). Association of adult body mass Index and Height with Anxiety, Depression, and Suicide in the General Population. The HUNT study. Am. J. Epidemiol..

[19] Stordal E, Bjelland I, Dahl AA, Mykletun A (2003). Anxiety and depression in individuals with somatic health problems. The Nord-Trøndelag Health Study (HUNT). Scand. J. Prim. Health Care.

[20] Brennan C, Worrall-Davies A, McMillan D, Gilbody S, House A (2010). The Hospital Anxiety and Depression scale: A diagnostic meta-analysis of case-finding ability. J. Psychosom. Res..

[21] Wu Y (2021). Accuracy of the Hospital Anxiety and Depression scale Depression subscale (HADS-D) to screen for major depression: Systematic review and individual participant data meta-analysis. BMJ.

[22] Leiknes KA, Dalsbø TK, Siqveland J (2016). Psychometric Assessment of the Norwegian Version of the Hospital Anxiety and Depression Scale.

[23] Mykletun A, Stordal E, Dahl AA (2001). Hospital Anxiety and Depression (HAD) scale: Factors structure, item analyses and internal consistency in a large population. Br. J. Psychiatry.

[24] Berk M (2013). So depression is an inflammation disease, but where does the inflammation come from?. BMC Med..

[25] White SL, Dunstan DW, Polkinghorne KR, Atkins RC, Cass A, Chadban SJ (2011). Physical inactivity and chronic kidney disease in Australian adults: The AusDiab study. Nutr. Metab. Cardiovasc. Dis..

[26] Bello AK (2007). Impact of weight change on albuminuria in the general population. Nephrol. Dial. Transplant..

[27] Bonnet F (2006). Waist circumference and the metabolic syndrome predict the development of elevated albuminuria in nondiabetic subjects: The DESIR Study. J. Hypertens..

[28] Andronico G (2005). Renal plasma flow, filtration fraction and microalbuminuria in hypertensive patients: Effects of chronic smoking. Nephrology.

[29] Warmoth L, Regalado MM, Simoni J, Harrist RB, Wesson DE (2005). Cigarette smoking enhances increased urine albumin excretion as a risk factor for glomerular filtration rate decline in primary hypertension. Am. J. Med. Sci..

[30] MacGiollabhui N, Alloy LB, Schweren LJS, Hartman CA (2021). Investigating whether a combination of higher CRP and depression is differentially associated with worse executive functioning in a cohort of 43,896 adults. Brain Behav. Immunity.

[31] Alexopoulos GS, Meyers BS, Young RC, Campell S, Silbersweig D, Charlson M (1997). "Vascular depression" hypothesis. Arch. Gen. Psychiatry.

[32] Krishnan KR, Hays JC, Blazer DG (1997). MRI-defined vascular depression. Am. J. Psychiatry.

[33] Gabin JM, Romundstad S, Saltvedt I, Holmen J (2019). Moderately increased albuminuria, chronic kidney disease and incident dementia: The HUNT study. BMC Nephol..

[34] Horn J, Romundstad S, Ellekjær H, Janszky I, Horn J (2020). Low grade albuminuria as a risk factor for subtypes of stroke—the HUNT Study in Norway. BCM Neurol..

[35] Hallan H, Romundstad S, Kvenild K, Holmen J (2003). Microalbuminuria in diabetic and hypertensive patients and the general population—consequences of various diagnostic criteria - the Nord-Trøndelag Health Study (HUNT). Scand. J. Urol. Nephrol..

[36] Brinkman J (2007). Prolonged frozen storage of urine reduces the value of albuminuria for mortality prediction. Clin. Chem..

[37] Bakker AJ (1999). Detection of microalbuminuria. Receiver operating characteristic curve analysis favors albumin-to-creatinine ratio over albumin concentration. Diabetes Care.

[38] Jensen JS, Clausen P, Borch-Johnsen K, Jensen G, Feldt-Rasmussen B (1997). Detecting microalbuminuria by urinary albumin/creatinine concentration ratio. Nephrol. Dial. Transplant..

[39] Bjerkeset O, Romild U, Smith GD, Hveem K (2011). The associations of high levels of C-reactive protein with depression and myocardial infarction in 9258 women and men from the HUNT population study. Psychol. Med..

[40] Lamers F, Vogerlzangs N, Merikangas KR, de Jonge P, Beekman ATF, Penninx BWJH (2013). Evidence for a differential role of HPA-axis function, inflammation and metabolic syndrome in melancholic versus atypical depression. Mol. Psychiatry.

[41] Hamer M, Batty G, Seldenrijk A, Kivimaki M (2011). Antidepressant medication use and future risk of cardiovascular disease: The Scottish Health Survey. Eur. Heart J..

[42] Nezafati MH, Eshraghi A, Vojdanparast M, Abtahi S, Nezafati P (2016). Selective serotonin ruptake inhibitors and cardiovascular events systematic review. J. Res. Med. Sci..

[43] Ibsen H (2005). Reduction in albuminuria translates to reduction in cardiovascular events in hypertensive patients: Losartan intervention for endpoint reduction in hypertension study. Hypertension.

[44] Sandhu S, Wiebe N, Fried LF, Tonelli M (2006). Statins for improving renal outcomes: A meta-analysis. J. Am. Soc. Nephrol..

[45] Asselbergs FW (2004). Prevention of renal and vascular endstage disease intervention trial (PREVEND IT) investigators. Effects of fosinopril and pravastatin on cardiovascular events in subjects with microalbuminuria. Circulation.

[46] Krokstad S (2013). Cohort profile: The HUNT study, Norway. Int. J. Epidemiol..

[47] Holmen J (2003). The Nord-Trøndelag Health Study 1995–97 (HUNT 2): Objectives, contents, methods and participation. Norsk Epidemiol..

