# Supplementary material for: Association between depression symptoms and moderately increased levels of the inflammation marker albuminuria is explained by age and comorbidity
Source: Sci Rep. 2022 May 25;12:8828. doi: 10.1038/s41598-022-12635-1 (PMC9132899; doi:10.1038/s41598-022-12635-1)
Supplement: Supplementary file 1 — Supplementary Information. [file 41598_2022_12635_MOESM1_ESM.docx]

**Supplementary Table 1.** Odds ratio (OR) for moderately increased albuminuria (ACR > 3.0 mg/mmol) according to different HADS-D subgroups/scores. Multivariable adjusted for age, sex (in the total population), systolic blood pressure, waist circumference, cholesterol, creatinine, cardiovascular disease, daily smoking of cigarettes, treated hypertension, diabetes, education, and hard physical activity, and with the interaction terms age*HADS-D and sex*HADS-D (total sample). ACR; albumin/creatinine ratio.

|  | **TOTAL** | | | **MEN** | | | **WOMEN** | | | |
| --- | --- | --- | --- | --- | --- | --- | --- | --- | --- | --- |
|  | **OR** | **95 % CI** | **P value** | **OR** | **95 % CI** | **P value** | **OR** | **95 % CI** | **P value** | |
| **HADS-D > 8**  Unadjusted  Adjusted for age (and sex)  + Interaction  Age*HADS (p=0.044)  Sex*HADS (p=0.358)  Multivariable adjusted  + Interactions  Age*HADS (p=0.045)  Sex*HADS (p=0.316) | 1.27  1.14  3.50  1.03  0.95  4.10  0.78 | 1.05-1.54  0.94-1.38  1.16-10.5  0.77-1.38  0.71-1.26  0.97-17.38  0.47-1.27 | 0.013  0.186  0.026  0.842  0.700  0.056  0.309 | 1.31  1.23  5.33  1.02  4.73 | 1.02-1.69  0.95-1.60  1.23-23.10  0.71-1.47  0.74-30.10 | 0.035  0.116  0.025  0.902  0.100 | 1.20  1.05  2.17  0.83  4.23 | 0.90-1.60  0.79-1.41  0.39-12.18  0.50-1.36  0.37-48.63 | 0.207  0.731  0.379  0.450  .  0.247 |  |
| **HADS-D > 11**  Unadjusted  Adjusted for age (and sex)  + Interactions  Age*HADS (p=0.571)  Sex*HADS (p=0.990)  Multivariable adjusted  + Interactions  Age*HADS (p=0.766)  Sex*HADS (p=0.835) | 1.59  1.46  0.86  1.46  0.97  1.41  0.91 | 1.19-2.14  1.08-1.98  0.13-5.59  0.94-2.29  0.61-1.54  0.12-17.31  0.40-2.05 | 0.002  0.014  0.873  0.094  0.894  0.788  0.807 | 1.49  1.46  0.71  0.94  0.59 | 1.00-2.22  0.96-2.21  0.05-9.26  0.53-1.68  0.02-17.05 | 0.052  0.074  0.793  0.833  0.756 | 1.69  1.49  1.21  0.95  7.74 | 1.09-2.62  0.96-2.33  0.07-20.45  0.42-2.16  0.18-325.1 | 0.018  0.077  0.894  0.896  0.283 | |
| **HADS-D continuous**  Unadjusted  Adjusted for age (and sex)  + Interactions  Age*HADS (p=0.819)  Sex*HADS (p=0.452)  Multivariable adjusted  + Interactions  Age*HADS (p=0.406)  Sex*HADS (p=0.559) | 1.05  1.02  1.04  1.01  1.00  1.07  0.98 | 1.03-1.07  1.00-1.05  0.92-1.18  0.98-1.05  0.97-1.03  0.91-1.26  0.93-1.04 | <0.001  0.036  0.552  0.405  0.845  0.436  0.559 | 1.05  1.03  1.07  1.00  1.09 | 1.02-1.08  1.00-1.06  0.90-1.27  0.96-1.04  0.88-1.35 | 0.001  0.051  0.424  0.922  0.441 | 1.04  1.02  1.01  1.00  1.07 | 1.01-1.08  0.99-1.05  0.84-1.22  0.94-1.05  0.83-1.39 | 0.007  0.280  0.909  0.849  0.583 | |

**Supplementary Table 2.** Odds ratio (OR) for moderately increased albuminuria (ACR > 3.0 mg/mmol) according to different HADS-A subgroups/scores. Multivariable adjusted for age, sex (in the total population), systolic blood pressure, waist circumference, cholesterol, creatinine, cardiovascular disease, daily smoking of cigarettes, treated hypertension, diabetes, education, and hard physical activity. ACR; albumin/creatinine ratio.

|  | **TOTAL** | | | **MEN** | | | **WOMEN** | | |
| --- | --- | --- | --- | --- | --- | --- | --- | --- | --- |
|  | **OR** | **95 % CI** | **P value** | **OR** | **95 % CI** | **P value** | **OR** | **95 % CI** | **P value** |
| **HADS-A > 8**  Unadjusted  Adjusted for age (and sex)  Multivariable adjusted | 0.88  0.97  0.77 | 0.70-1.10  0.77-1.23  0.54-1.08 | 0.264  0.809  0.123 | 0.92  1.03  0.80 | 0.66-1.27  0.74-1.45  0.51-1.26 | 0.595  0.846  0.342 | 0.92  0.93  0.72 | 0.67-1.28  0.67-1.29  0.43-1.21 | 0.629  0.667  0.214 |
| **HADS-A > 11**  Unadjusted  Adjusted for age (and sex)  Multivariable adjusted | 1.04  1.22  0.94 | 0.74-1.48  0.86-1.74  0.57-1.55 | 0.808  0.274  0.819 | 1.18  1.46  1.24 | 0.72-1.94  0.87-2.44  0.64-2.41 | 0.519  0.153  0.525 | 1.04  1.07  0.69 | 0.64-1.67  0.65-1.75  0.31-1.50 | 0.888  0.796  0.352 |
| **HADS-A continuous**  Unadjusted  Adjusted for age (and sex)  Multivariable adjusted | 0.97  0.99  0.96 | 0.94-0.99  0.96-1.01  0.93-1.00 | 0.006  0.316  0.046 | 0.98  1.01  0.98 | 0.95-1.01  0.97-1.04  0.93-1.03 | 0.218  0.736  0.362 | 0.96  0.97  0.95 | 0.93-1.00  0.93-1.01  0.90-1.00 | 0.040  0.099  0.053 |

**Supplementary Table 3.**

Basic characteristics of the responders versus the non-responders on HADS-D-questions.

Values are mean (+SD) unless otherwise stated. N.s, non-significant; ACR, albumin/creatinine ratio ; CVD, cardiovascular disease.

| **TOTAL** | **HADS-D**  n=7794 | **HADS-D missing**  n=509 | **P value** |
| --- | --- | --- | --- |
| **Age**, years  **Sex** (M %) | 60.6 (14.7)  46.7 | 66.4 (12.7)  41.1 | <0.001  0.014 |
| **ACR**, mg/mmol | 1.71 (3.08) | 1.78 (2.88) | N.s |
| **ACR > 3.0** mg/mmol (%) | 11.1 | 13.6 | N.s |
| **SBP**, mmHg | 150 (23) | 154 (23) | <0.001 |
| **DBP**, mmHg | 85 (13) | 86 (13) | N.s |
| **BMI**, kg/m2 | 28.3 (4.5) | 28.4 (4.5) | N.s |
| **Waist circumference**, cm | 91.4 (12.1) | 92.3 (12.1) | N.s |
| **Creatinine** mmol/l | 91.7 (17.7) | 92.6 (17.9) | N.s |
| **Cholesterol**, mmol/l | 6.2 (1.2) | 6.5 (1.3) | <0.001 |
| **Triglycerides**, mmol/l | 2.06 (1.27) | 2.16 (1.30) | N.s |
| **Glucose**, mg/dl | 6.2 (2.5) | 6.5 (2.5) | 0.022 |
| **Diabetes** (%) | 16.1 | 20.4 | 0.009 |
| **Smoking daily** (%) | 20.5 | 16.9 | 0.056 |
| **Education high** (%) | 13.0 | 7.2 | <0.001 |
| **Hard physical activity** (%) | 26.2 | 16.8 | 0.001 |
| **Treated hypertension** (%) | 64.4 | 74.0 | <0.001 |
| **CVD** (%) | 20.8 | 28.1 | <0.001 |

**Supplementary Table 4.** Mean, standard deviation (SD) and correlation matrix with the variables. Pearson correlation coefficients and p-values;

*< 0.01, **< 0.001, ***<0.0001. ACR; albumin/creatinine ratio, SBP; systolic blood pressure, BMI; body mass index, HT; hypertensive, CVD; cardiovascular disease

|  | **Mean** | **SD** | **1.** | **2.** | **3.** | **4.** | **5.** | **6.** | **7.** | **8.** | **9.** | **10.** | **11.** | **12.** | **13.** | **14.** | **15.** | **16.** | **17.** |
| --- | --- | --- | --- | --- | --- | --- | --- | --- | --- | --- | --- | --- | --- | --- | --- | --- | --- | --- | --- |
| **1. Sex** | 0.46 | 0.50 | 1 |  |  |  |  |  |  |  |  |  |  |  |  |  |  |  |  |
| **2. Age** | 61.0 | 14.7 | -0.060  ** | 1 |  |  |  |  |  |  |  |  |  |  |  |  |  |  |  |
| **3. ACR** | 1.72 | 3.07 | 0.074  ** | 0.173  ** | 1 |  |  |  |  |  |  |  |  |  |  |  |  |  |  |
| **4. SBP** | 150 | 23 | -0.054  ** | 0.458  *** | 0.169  ** | 1 |  |  |  |  |  |  |  |  |  |  |  |  |  |
| **5. Waist circumference** | 91.4 | 12.1 | 0.339  ** | 0.192  ** | 0.115  ** | 0.162  ** | 1 |  |  |  |  |  |  |  |  |  |  |  |  |
| **6. BMI** | 28.0 | 4.5 | -0.086  ** | 0.115  ** | 0.054  ** | 0.158  ** | 0.774  *** | 1  -- |  |  |  |  |  |  |  |  |  |  |  |
| **7. Cholesterol** | 6.3 | 1.2 | -0.180  ** | 0.304  ** | 0.028  * | 0.263  ** | 0.076  ** | 0.128  ** | 1 |  |  |  |  |  |  |  |  |  |  |
| **8. Triglycerides** | 2.06 | 1.27 | 0.067  ** | 0.127  ** | 0.109  ** | 0.136  ** | 0.303  ** | 0.259  ** | 0.317  ** | 1 |  |  |  |  |  |  |  |  |  |
| **9. Glucose** | 6.2 | 2.5 | 0.066  ** | 0.161  ** | 0.148  ** | 0.113  ** | 0.154  ** | 0.106  ** | -0.004 | 0.177  ** | 1 |  |  |  |  |  |  |  |  |
| **10. Creatinine** | 91.8 | 17.8 | 0.388  ** | 0.217  ** | 0.186  ** | 0.067  ** | 0.219  ** | 0.035  * | 0.039  ** | 0.137  ** | 0.055  ** | 1 |  |  |  |  |  |  |  |
| **11. HADS D** | 4.0 | 3.2 | 0.022 | 0.149  ** | 0.045  ** | 0.056  ** | 0.115  ** | 0.079  ** | 0.069  ** | 0.087  ** | 0.034  * | 0.062  ** | 1 |  |  |  |  |  |  |
| **12. Diabetes** | 0.16 | 0.37 | 0.037  ** | 0.130  ** | 0.142  ** | 0.059  ** | 0.137  ** | 0.092  ** | -0.038  ** | 0.116  ** | 0.571  *** | 0.016 | 0.062  ** | 1 |  |  |  |  |  |
| **13. Anti-HT**  **treatment** | 1.68 | 0.94 | 0.053  ** | -0.417  *** | -0.080  ** | -0.346  ** | -0.228  ** | -0.240  ** | -0.206  ** | -0.144  ** | 0.076  ** | -0.163  ** | -0.098  ** | 0.219  ** | 1 |  |  |  |  |
| **14. Smoking** | 0.20 | 0.40 | 0.016 | -0.173  ** | 0.012 | -0.135  ** | -0.101  ** | -0.149  ** | -0.028  * | 0.007 | -0.045  ** | -0.106  ** | 0.035  * | -0.041  ** | 0.122  ** | 1 |  |  |  |
| **15. CVD** | 0.21 | 0.41 | 0.122  ** | 0.311  ** | 0.118  ** | 0.051  ** | 0.108  ** | 0.012 | 0.040  ** | 0.105  ** | 0.077  ** | 0.196  ** | 0.123  ** | 0.058  ** | -0.211  ** | -0.052  ** | 1 |  |  |
| **16. Hard phys.**  **activity** | 0.26 | 0.44 | 0.141  ** | -0.247  ** | -0.068  ** | -0.130  ** | -0.091  ** | -0.103  ** | -0.153  ** | -0.103  ** | -0.041  * | -0.026 | -0.107  ** | -0.021 | 0.174  ** | -0.021 | -0.097  ** | 1 |  |
| **17. Education** | 0.13 | 0.33 | 0.069  ** | -0.212  ** | -0.046  ** | -0.169  ** | -0.071  ** | -0.080  ** | -0.108  ** | -0.074  ** | -0.048  ** | 0.016 | -0.083  ** | -0.033  * | 0.118  ** | -0.062  ** | -0.077  ** | 0.162  ** | 1 |
